# The Function of Root Exudates in the Root Colonization by Beneficial Soil Rhizobacteria

**DOI:** 10.3390/biology13020095

**Published:** 2024-02-02

**Authors:** Lin Chen, Yunpeng Liu

**Affiliations:** 1 National Permanent Scientific Research Base for Warm Temperate Zone Forestry of Jiulong Mountain, Experimental Center of Forestry in North China, Chinese Academy of Forestry, Beijing 102300, China; 2 State Key Laboratory of Efficient Utilization of Arid and Semi-Arid Arable Land in Northern China, The Institute of Agricultural Resources and Regional Planning, Chinese Academy of Agricultural Sciences, Beijing 100081, China

**Keywords:** rhizobacteria, root exudation, root colonization, root exudate–rhizobacteria interaction

## Abstract

**Simple Summary:**

This review is focused on the role of root exudates on beneficial rhizobacterial colonization. The dynamic interaction between root exudates and rhizobacteria is influenced by plant genotype, development, environmental biotic factors, and abiotic factors. Bacterial-specific metabolism plays crucial roles in rhizosphere competence and rhizobacterial colonization ability. Diverse types of root exudates serve as nutrients, signals, or antimicrobial substances, facilitating root colonization by beneficial bacteria. Certain secondary metabolites selectively promote root colonization by bacteria with specialized functions.

**Abstract:**

Soil-beneficial microbes in the rhizosphere play important roles in improving plant growth and health. Root exudates play key roles in plant–microbe interactions and rhizobacterial colonization. This review describes the factors influencing the dynamic interactions between root exudates and the soil microbiome in the rhizosphere, including plant genotype, plant development, and environmental abiotic and biotic factors. We also discuss the roles of specific metabolic mechanisms, regulators, and signals of beneficial soil bacteria in terms of colonization ability. We highlight the latest research progress on the roles of root exudates in regulating beneficial rhizobacterial colonization. Organic acids, amino acids, sugars, sugar alcohols, flavonoids, phenolic compounds, volatiles, and other secondary metabolites are discussed in detail. Finally, we propose future research objectives that will help us better understand the role of root exudates in root colonization by rhizobacteria and promote the sustainable development of agriculture and forestry.

## 1. Introduction

The rhizosphere, the narrow zone of soil influenced by the plant root system, is a dynamic environment where complex interplay between plants and soil microbes occurs, and it may contain up to 10^11^ cells/g of root, with more than 30,000 bacterial species [1]. There are various signals in the rhizosphere, including QS signals among microorganisms and root exudate signals from plants to microorganisms [2]. Root exudates are a mixture of organic compounds released by plant roots. Plants can change the soil environment, shape the rhizosphere microbiome, and improve their growth conditions through root exudation [3]. Diverse root exudates shape the distinct rhizosphere environment and microbiome. Soil conditions affected by root exudates regulate plant–soil feedback, while the rhizosphere microbiome plays a key role in plant–soil feedback [4]. The rhizospheric microbiome is always distinct from the bulk soil microbiome. This phenomenon, known as the rhizosphere effect, is influenced by metabolites in the rhizosphere. The strength of the rhizosphere effects varies by plant species. Some plant species, such as maize and lotus, exhibit strong rhizosphere effects [5,6,7], while other plant species, such as rice and *Arabidopsis*, exhibit weak rhizosphere effects [8,9]. 

**Figure 1:**
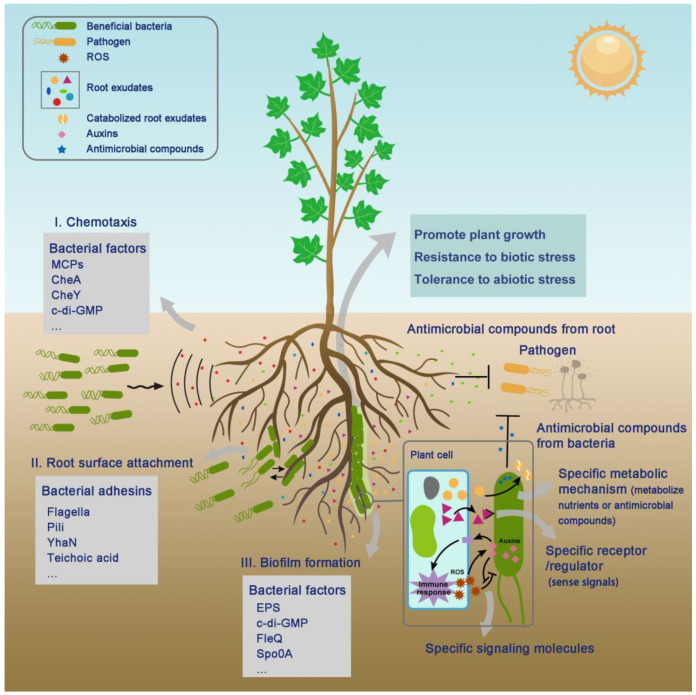
Colonization process of beneficial bacteria in the rhizosphere. Colonization by nonsymbiotic beneficial rhizobacteria consists of several steps, including chemotaxis, root surface attachment, and biofilm formation [11]. Root exudates serve as signals, nutrients, and antimicrobial compounds during the colonization process. The receptors and regulators from bacteria are represented in the gray boxes. The specific metabolic mechanisms, regulators, and signals of beneficial bacteria and tolerance to antimicrobial compounds determine the colonization ability of beneficial rhizobacteria. Successful colonization by beneficial bacteria provides various benefits to plants.

Root exudates facilitate communication with soil microorganisms, which in turn affects plant health through various pathways, such as enriching beneficial microbes. The enriched beneficial microbes in the rhizosphere play key roles in plant health, productivity, and fitness. Beneficial rhizobacteria, generally called plant growth-promoting rhizobacteria (PGPRs), can promote plant growth and health by providing numerous benefits and are widely used in agricultural production, such as *Bacillus* spp. and *Pseudomonas* spp. [10,11]. They can facilitate nutrient acquisition by fixing atmospheric nitrogen, mobilizing soil phosphorus, and solubilizing minerals [12,13]. Furthermore, they can produce growth-promoting substances that enhance plant growth and development, such as phytohormones and enzymes [13,14]. Some beneficial rhizobacteria can also suppress plant diseases by producing antimicrobial compounds, competing with pathogens for resources, and inducing systemic resistance [15,16]. Additionally, they help plants cope with abiotic stress by inducing systemic tolerance [17] (Figure 1). In this review, we focus on the roles of root exudates in the colonization process of beneficial soil rhizobacteria.

Successful root colonization and niche adaptation are essential for rhizobacteria to exert beneficial effects on plants. In general, the process of soil rhizobacterial colonization begins with chemotaxis followed by root surface attachment and biofilm formation, which has been well researched recently [11]. Chemotaxis is the first step in the root colonization by motile bacteria (Figure 1). Currently, most of the chemoattractants in root exudates identified for rhizobacteria are low-molecular-weight compounds. Methyl-accepting chemotaxis proteins (MCPs), CheA histidine kinase, and the coupling regulator CheY are the key components of the chemosensory pathway [11,18]. Attachment to the plant host can be classified as primary attachment or secondary attachment based on the strength of the interaction between bacteria and plants. The process of attaching to plant roots is mediated by various bacterial adhesins, such as flagella, pili, fimbriae [19], cellulose fibrils [20], extracellular proteins [21], exopolysaccharides (EPSs) [22], and lipopolysaccharides (LPSs) [23]. Biofilms are complex and structured communities of microorganisms embedded in a self-produced matrix that allows them to establish stable and long-term associations with plants. The matrix is composed of water-soluble polysaccharides; extracellular DNA amyloids; proteins; and water-insoluble substances, such as lipids, amyloids, cellulose, and humic-like refractories [24]. Once biofilms form, rhizobacteria can access the nutrient-rich microenvironment within the biofilm. Additionally, biofilms can act as protective barriers against biotic and abiotic stresses, such as desiccation, nutrient limitation, antimicrobial compound exposure, and the presence of other competitive microorganisms [25].

Root exudates play a vital role in promoting colonization by soil rhizobacteria. Plants selectively recruit specific microbes to the rhizosphere. Certain compounds in root exudates act as signaling molecules that mediate motility, chemotaxis, biofilm formation, the “cry-for-help” response, and symbiotic relationships [11,26]. In addition, this colonization process is influenced by the unique composition of nutrients and antimicrobial compounds in root exudates, which promote the growth and proliferation of bacteria and outcompete pathogenic microbes [27,28] (Figure 1).

The interplay between root exudates and soil microbial colonization is a cornerstone of plant–microbe symbiosis and contributes to the overall fitness and productivity of plants. Successful colonization can lead to closer associations between plants and microbes, which is necessary for beneficial bacteria to perform their beneficial functions. However, the effects of dynamic root exudates and beneficial bacterial-specific mechanisms on root colonization have not been comprehensively concluded. The aim of this review is to summarize the recent advances in understanding the influencing factors of dynamic interactions between root exudates and beneficial rhizobacteria in the soil rhizosphere. Additionally, we will review the advances in characterizing the specific metabolic mechanisms employed by beneficial bacteria in rhizosphere competence and root colonization. Finally, we summarize the progress in research on the function of diverse types of root exudates in facilitating root colonization by beneficial soil bacteria. A comprehensive understanding of the interaction between root exudates and beneficial bacteria during root colonization, as well as the key root exudates that promote beneficial bacterial root colonization, will contribute to the application of beneficial rhizobacteria in agriculture and forestry.

## 2. Composition of Root Exudates and Influencing Factors of Plants and the Environment on the Rhizosphere

Numerous studies have shown that root exudates can attract beneficial microorganisms, shape the rhizosphere microbiome, and improve plant performance [3,11]. In general, plants release 11–40% of their photosynthetic yield into the rhizosphere in the form of root exudates [15], which can be categorized as low- or high-molecular-weight compounds. Low-molecular-weight compounds encompass diverse molecules, such as sugars, organic acids, amino acids, alcohols, volatile compounds, and other secondary metabolites. High-molecular-weight compounds, such as mucilage (polysaccharides) and proteins, may exhibit less variability but usually constitute a larger fraction of exudates [28]. Most low-molecular-weight compounds, such as sugars, amino acids, and ions, are passively released via diffusion, channel, and vesicle transport. For example, the efflux of sugars is facilitated by “sugars will eventually be exported transporters” (SWEETs) along a concentration gradient [29,30]. However, other compounds, such as secondary metabolites, proteins, and polysaccharides, are generally secreted into the rhizosphere by an active mechanism via different membrane-bound proteins. For example, secondary metabolites, such as flavonoids, phenolics, and hormones, are actively secreted into the rhizosphere with the help of ATP-binding cassette (ABC) transporters or multidrug and toxic compound extrusion (MATE) transporters [26,31]. Notably, citrate, a low-molecular-weight compound, is also transported by MATE antiporters [32].

### 2.1. Effects of Plant Genotype and Development on Root Exudate–Rhizobacteria Interactions

**Figure 2:**
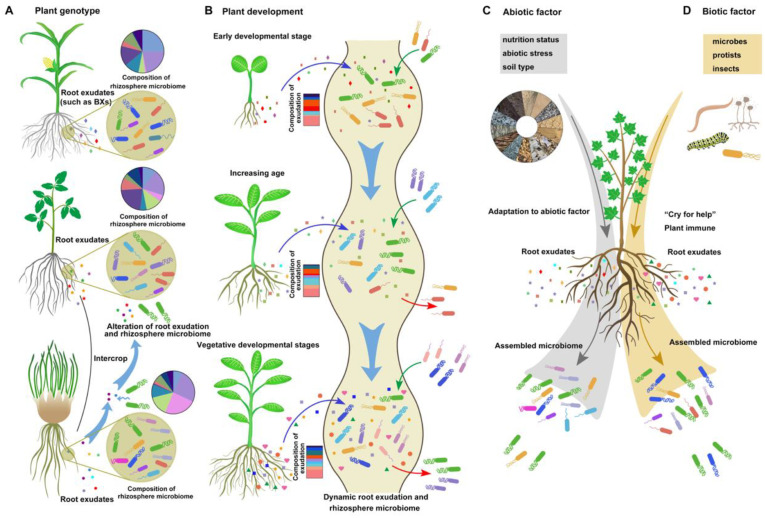
The influencing factors on root exudation and rhizosphere bacteria composition. The root exudation–rhizobacteria interaction is a dynamic process impacted by plant genotype (**A**), plant development (**B**), environmental abiotic factors (**C**), and biotic factors (**D**): (**A**) The process of root exudation displays variability across different plant genotypes. The diverse signaling receptors of bacteria and their ability to utilize and decrypt substances contribute to changes in the rhizosphere microbiome in the rhizosphere with different compositions of root exudates. (**B**) The blue arrow represents dynamic root exudation at different plant developmental stages. The green and red arrows indicate the attraction and repulsion of rhizobacteria, respectively. (**C**,**D**) In response to abiotic or biotic stress, plants can recruit some beneficial bacteria through exudation.

The composition and quantity of root exudates are not static or uniform and change dynamically based on several factors. First, root exudates are determined by plant genotype (Figure 2A). As the distance between microorganisms and roots decreases (from rhizosphere soil to the endorhizosphere), the influence of plant genotype on the enrichment of microorganisms increases [33]. Many metabolites are shared across different species, while others are specific to certain plant species. For instance, benzoxazinoids (BXs), a class of defensive secondary metabolites, are predominantly released by the crown roots of different grass species, such as maize and wheat [3]. In addition, rhizobacteria exhibit diverse responses to root exudates from various plant genotypes [34]. This phenotypic trait allows the use of an intercropping system to improve the rhizosphere environment and establish a healthier microbial community. A recent study on an intercropping system involving tomato and potato onion revealed distinct exudation patterns for each species. The presence of raxifolin in potato onion root exudates alters the chemistry of tomato root exudates, leading to the recruitment of beneficial soil bacteria, such as *Bacillus* sp., and the establishment of a healthy rhizosphere [35] (Figure 2A).

Furthermore, the composition of root exudates is influenced by plant diversity. High plant biodiversity is typically associated with high microbial diversity due to diverse exudates [26]. Essarioui et al. [36,37] reported that *Streptomyces* isolates, which are well known for producing antibiotics, from soil with polyculture plant species have larger niche widths than those from monoculture soil. In contrast, pathogenic *Fusarium* exhibits a greater niche preference in monocultures than in polycultures. The differences in the utilization of diverse exudates by beneficial or pathogenic bacteria may contribute to this phenomenon. Leveraging this phenomenon, we can potentially manipulate the soil microbial community by adopting different cropping patterns.

Second, root exudates are dynamic and change according to plant development (Figure 2B). A recent study demonstrated that *Avena barbata* produced higher levels of sucrose and homoserine during the early developmental stage than during other stages. With increasing age, the exudation of amino acids and carboxylic acids increases. At the vegetative development stage, *A. barbata* exhibited the greatest overall root exudation. During plant senescence, the abundance of quaternary ammonium salts and hormones significantly increases [15]. Similarly, in *Arabidopsis*, the highest abundance of sugars occurs at the early stage, predominantly used for root growth maintenance, while amino acids and phenolic exudation increase at the late plant developmental stage [38,39]. The rhizosphere microbial community and function are influenced by altered root exudates throughout different plant developmental stages [38].

Plants adjust their exudation patterns at different developmental stages to regulate the rhizosphere microbiome and fulfill the nutrient requirements of different growth periods [39]. Moreover, different bacterial communities are recruited by plants during various developmental stages, reflecting distinct choices in root exudate utilization (Figure 2B). Different plant species and development can selectively enrich specific microbial taxa that have the right transporters and enzymes to metabolize those specific root exudates, thereby shaping the microbiome composition. For instance, isolates recruited by *A. barbata* during the root growth stage display a preference for sugars, amino acids, organic acids, and quaternary amines [15]. Consequently, the nutrient preferences of bacterial strains lead to the enrichment of specific microbiome compositions at different stages of plant development.

### 2.2. Effects of Environmental Abiotic and Biotic Factors on Root Exudate–Rhizobacteria Interactions

Third, exudation is modulated by various environmental abiotic factors, including nutrient status, soil type, and abiotic stressors (Figure 2C). Nutrient deficiencies or excesses can alter the composition and quality of root exudates, potentially impacting the selection and growth of rhizobacterial communities. For example, coumarins, which are always induced by iron starvation, are phenolic secondary metabolites produced in roots and exuded into the rhizosphere in a MYB72/BGLU42-dependent manner in *Arabidopsis* [40]. Coumarins are necessary for beneficial interactions between *Arabidopsis* plants and the root microbiota under iron starvation conditions and can modulate the root microbial community, elicit microbe-associated iron nutrition, improve plant iron nutrition, and aid in the health of the soil rhizosphere [41]. The availability and composition of nutrients in the rhizosphere are also determined by the soil structure, such as the soil matrix and soil aggregates [42]. A rhizosphere with high nutrient availability may increase microbial carbon metabolism and facilitate the development of complex networks of soil rhizosphere microorganisms [43]. Soil pH, an important characteristic of soil type, plays a crucial role in determining the availability and effectiveness of root exudates, as well as the survival and growth of rhizobacteria [44,45]. Wan et al. [44] reported that soil pH has a greater influence on rhizobacterial communities than other physicochemical variables and vegetation types. Guo et al. [46] suggested that the soil microbial network in the rice rhizosphere becomes more stable and complex at higher pH levels (pH > 7.5), which is associated with improved efficiency of nutrient cycling efficiency. Additionally, plants can modulate soil pH by regulating the composition of root exudates, thereby affecting the presence of beneficial and pathogenic bacteria [45].

Various abiotic stressors, such as salinity, heavy metals, pollutants, and drought, substantially influence root exudate–rhizobacteria interactions. These stressors can modify root exudation patterns, impair the viability of rhizobacteria, and disrupt the symbiotic relationship between plants and rhizobacteria [47]. For instance, salt stress can lead to the dominance of certain organic acids as major components in the root exudates of the halophyte *Limonium sinense*, promoting the growth and chemotaxis of the beneficial strain *B. flexus* KLBMP 4941 and subsequently benefiting plant growth under salt stress [48]. Under heavy metal stress, more exudates, particularly organic nitrogen, are released to attract the beneficial bacterium *P. putida* E36, which aids *Miscanthus* × *giganteus* plants in coping with heavy metal stress [49]. The abundance of organic acids and amino acids in rice exudates increased in response to chlorpyrifos stress, attracting the beneficial soil bacterium *Sphingomonas* sp. to degrade chlorpyrifos [50]. In addition, soil moisture levels are the key factors affecting the quantity and quality of root exudation and rhizobacterial colonization [51]. For instance, drought stress leads to increased production of many metabolites secreted by roots, with a notable increase in glycerol-3-phosphate. Altered plant metabolites enhance the abundance and activity of monoderm bacteria, which in turn improve plant fitness under drought stress [52]. By considering and managing these abiotic factors, we can enhance the efficiency and effectiveness of rhizobacterial colonization and maximize the benefits derived from this symbiotic relationship.

Fourth, exudation is influenced by various environmental biotic factors. Specific changes in root exudation patterns can be induced by different microbes, protists, and insects, such as many secondary metabolites and small peptides (Figure 2D). Differential immune and metabolic responses occur in roots when these plants interact with beneficial and pathogenic microbes, leading to either the promotion or suppression of colonization. A recent study showed that glycosylated azelaic acid, which is induced by the soil microbiome, may act as a potential microbe-induced signal in reprogramming systemic root exudates [53]. Some altered compounds in root exudates exhibit variable antimicrobial activity and can function as plant bioprotectants against pathogens and as attractants for certain plant-associated bacteria [28]. Increasing evidence indicates that upon colonization by beneficial bacteria, plants activate specific immune responses, while certain beneficial bacteria exhibit mechanisms to tolerate root immune responses through spatial evasion and high tolerance strategies [11]. The immune response activated by the beneficial strain *Pseudomonas* varies at different sites in the root, suggesting that the spatial mitigation of the colonization site may contribute to *Pseudomonas* sp. evasion of root immunity [54,55]. The PGPR *B. velezensis* SQR9 can tolerate the bursts of ROS through the specific two-component regulatory system ResDE [56]. Additionally, upon colonization by beneficial bacteria, the presence of many compounds exuded into root exudates, such as phenolics, acyl sugars, and some antimicrobial compounds, changes to improve beneficial bacterial colonization and inhibit pathogen colonization [53,57,58].

After pathogen infection or herbivore damage, plants can “cry for help” with root exudates and regulate plant immune signaling. The altered composition of root exudates caused by pathogens or herbivores leads to an increase in antimicrobial substances specific to the pathogen, as well as chemoattractants that attract beneficial rhizobacteria. For instance, upon infection by the fungal pathogen *Fusarium culmorum*, the blend of VOCs emitted by *Carex arenaria* roots changes, and some specific bacteria with antifungal properties are attracted. Among the altered VOCs, several substances, such as benzofuran and acetophone, exhibit antifungal activity [59]. Furthermore, the infection of ginger by the pathogen *Ralstonia solanacearum* enhances the secretion of several antibacterial compounds by gingers and increases the abundance of beneficial and stress-tolerant bacteria, ultimately inhibiting ginger bacterial wilt [60]. In potato plants, aphid infection reduces the glucose and fructose levels in root exudates [61]. In addition, the phytohormone salicylic acid not only induces plant immune signaling in response to pathogens but also modulates colonization by specific rhizobacteria [62]. These findings support the notion that some defense signals activated by pathogen infection might promote some beneficial bacterial colonization.

## 3. The Specific Mechanisms That Determine the Ability of Beneficial Bacteria to Colonize Plants

### 3.1. Specific Metabolic Mechanisms

The capacity to utilize or metabolize substances from root exudates is critical for soil rhizosphere bacteria to occupy rhizosphere niches (Figure 1). Zhalnina et al. [15] reported that the uptake of most root exudates, including a large proportion of amino acids, nucleotides, and sugars, by soil rhizosphere bacteria is similar. This is because the uptake patterns of some metabolites from the same class are similar across isolates. Nevertheless, there is considerable variability among soil rhizosphere bacteria concerning the absorption of certain compounds, such as specific organic acids, fatty acids, and quaternary amines. The division of the rhizosphere ecological niche is based on the utilization of the root exudate substrate by different rhizosphere bacteria. For example, Huang et al. [63] discovered that the specialized triterpenes thalianin, thalianyl fatty acid esters, and arabidin in *Arabidopsis* can serve as carbon sources and moderate the root microbiota by affecting the growth of specific bacterial taxa. Root bacteria that can selectively metabolize certain triterpenes as carbon sources for growth have greater rhizosphere competence [63]. Furthermore, the preference and competitiveness of rhizosphere bacteria for root exudates can be predicted from genome sequence information. Bacteria with a greater number of transporters for specific nutrients tend to absorb resources more rapidly, enabling them to outcompete others in the rhizosphere [15]. For example, an increase in transporter activity, such as that of ABC transporters, was correlated with changes in root metabolite profiles and was considered to be associated with the proliferative advantage observed in certain bacteria, such as monoderm bacteria [52]. A recent study showed that a putative ABC transporter (YhaN) in *B. velezensis* SQR9 also serves as an adhesin during the colonization and functions on both cucumber root surfaces and abiotic surfaces [64]. Additionally, 1-aminocyclopropane-1-carboxylic acid (ACC) in root exudates can be used only by bacteria with ACC deaminase. These bacteria can degrade ACC as a nitrogen source, giving them a significant advantage in terms of rhizosphere competitiveness [14]. In addition, plant nutrients can be decrypted by select microbes. Glycans, abundant in root exudates [52], can function in encrypting nutrients through glycosylation, with only certain bacteria capable of decrypting them to establish dominance within the rhizosphere [65].

### 3.2. Specific Receptors

Many root exudates serve as crucial signals in plant–rhizobacteria interactions, enhancing bacterial colonization in a dose-dependent manner through processes such as chemotaxis, root surface attachment, and biofilm formation [13] (Figure 1). The range of molecules sensed by a specific strain is determined by the MCP type. Different receptors, such as McpA, McpB, McpC, and TlpB, which have been identified as mediators of chemotaxis to various compounds, are associated with the recognition of specific substances due to their varying structures [18]. To adapt to diverse environmental conditions, efficient colonizers in the rhizosphere must be able to respond effectively to a wide variety of compounds. For example, certain PGPRs, such as *B. velezensis* SQR9, *P. fluorescens* Pf0-1, and *P. putida* KT2440, exhibit a broad response to various compounds in root exudates through their diverse receptors [18,66,67,68]. In addition to MCPs, many regulators have been found to play important roles in root colonization by beneficial soil bacteria. For instance, the global regulator FleQ, a multimeric cyclic diguanylate binding protein, was recently shown to regulate flagellar motility and the expression of biofilm matrix components in *Pseudomonas* [69]. Additionally, the bacterial second messenger c-di-GMP has been demonstrated to play a crucial role in the chemotaxis response and biofilm formation of several motile bacteria, such as *Bacillus velezensis*, *Azospirillum brasilense*, and *Pseudomonas putida* [24,70,71]. Upon binding c-di-GMP to FleQ, FleQ is transformed from a repressor to an activator [72]. In another well-studied beneficial rhizobacterium, *Bacillus* spp., biofilm formation is governed by two master transcription factors, Spo0A and DegU [73,74].

### 3.3. Specific Signaling Molecules

Furthermore, the specific signaling molecules produced by bacteria are also important for root colonization (Figure 1). Phytohormones, particularly auxins, are both produced and degraded by many root-colonizing bacteria, contributing to root colonization and the formation of complex bacteria–plant communication networks [75]. Auxin inhibits root growth at high concentrations [76]. Certain species from the genus *Variovorax* can both produce and degrade auxin to finely regulate auxin concentrations in the *Arabidopsis* rhizosphere, with the MarR family of regulators in the genus *Variovorax* playing key roles in auxin degradation [77]. In the case of cucumber roots colonized by pathogenic fungi or the beneficial strain *B. velezensis* SQR9, the amount of tryptophan secreted by the roots is enhanced. An elevated tryptophan level contributes to the production of indole-3-acetic acid (IAA) by SQR9 and aids in colonization by SQR9 [78]. Furthermore, the enhanced production of IAA by SQR9 may also support root colonization. Tzipilevich et al. [79] discovered that, aside from promoting plant growth, bacterial auxin plays an important role in root colonization by the beneficial bacterium *B. velezensis* FZB42 by counteracting the plant immune response in an EFR-dependent manner and ROS toxicity. Root colonization by bacteria triggers a plant immune response in which the ROS production by the plant immune system further stimulates bacterial auxin secretion to mitigate ROS toxicity, promoting bacterial survival and colonization. Interestingly, auxin also induces the expression of the plant immune receptor EFR, thereby accelerating the feedback loop. Moreover, in pathogens, bacterial auxin can also inhibit the plant immune response through antagonistic interactions with the SA signaling pathway to facilitate pathogen infection [80,81].

## 4. The Colonization-Regulating Compounds in the Root Exudates

Numerous substances in root exudates serve as energy sources, nutrient supplies, and communication signals for both plants and rhizosphere microorganisms and attract or repel certain strains [11]. Here, we will focus on specific compounds, including organic acids, amino acids, sugars, sugar alcohols, flavonoids, phenolic compounds, volatiles, and other secondary metabolites (Table 1), as nutrients, signals, or antimicrobial substances involved in the colonization process of beneficial soil rhizobacteria.

### 4.1. Organic Acids

Different types of organic acids, such as malic acid, critic acid, fumaric acid, tartaric acid, and succinic acid, are reported to be nutrients for microbes and signals during the process of colonization by diverse beneficial soil rhizobacteria [4,26]. Organic acid treatment has been shown to improve soil physicochemical conditions and alter the soil microbial community, especially by enhancing colonization by some beneficial bacteria by inducing the growth, chemotactic response, motility, and biofilm formation of rhizobacteria [101,102]. For example, the biofilm formation of *Hansschlegelia zhihuaiae* S113 is mediated by fumaric acid, tartaric acid, and L-malic acid through the regulation of the expression of motility/chemotaxis (fla/che cluster) proteins, and the resulting biofilm facilitates bensulfuron-methyl degradation [82]. Oxalate can be metabolized by plant-associated *Burkholderia* species as a carbon source [103] but cannot be utilized by plant or human pathogenic strains [83]. These results support the hypothesis that compounds selectively used by specific bacteria play a critical role in the competitive dynamics of rhizosphere niches and in facilitating communication between plants and rhizobacteria (Figure 2). Additionally, the colonization and growth-promoting functions of different beneficial bacteria induced by the same substance are similar and specific, even for different strains in the same genus. For instance, in rice roots, inoculation with the PGPR *B. altitudinis* LZP02 induces the secretion of citric acid, which in turn enhances chemotaxis, biofilm formation, and the growth-promoting ability of *B. altitudinis* LZP02 [84]. However, in the rhizosphere interaction of peanut plants and the beneficial *Burkholderia pyrrocinia* strain P10, several organic acids (including citric, malic, and oxalic acid) promoted bacterial biofilm formation, while the growth-promoting ability of the strain was stimulated by specific amino acids rather than organic acids [13]. 

### 4.2. Amino Acids

Amino acids play an important role in root exudates, as they serve as a primary source of nitrogen for many soil microbes and promote the growth and proliferation of beneficial microbes. These amino acids can be directly utilized by microbial communities to build their own proteins or can be catabolized for energy [104]. Specific amino acids in exudates can act as attractants for beneficial soil rhizobacteria and selectively enhance the growth of beneficial rhizobacteria over pathogenic rhizobacteria [26]. For example, histidine, arginine, and aspartate, which are sensed by TlpH, play important roles in the chemotactic response of the beneficial strain *Azorhizobium caulinodans* ORS571 [85]. Furthermore, several amino acids act as attractants for *P. fluorescens* Pf0-1, with CtaA, CtaB, and CtaC acting as the receptors for sensing amino acids [86]. However, amino acids can also serve as carbon and nitrogen sources and are likely positively related to the abundance of opportunistic pathogenic bacteria capable of amino acid uptake [4]. For example, tyramine and other amino acids secreted by tomato roots promote the growth of the pathogen *Spongospora subterranea* [105].

### 4.3. Sugars

Next, we will focus on the impact of sugars on the colonization by plant-beneficial Gram-positive bacteria, specifically *Bacillus* strains. Sugars act as the main carbon source and colonization signals for rhizobacteria [26]. Sucrose, a ubiquitous disaccharide that is abundantly secreted by roots, selectively shapes the soil microbial community, particularly that of bacilli and pseudomonads [87]. *B. subtilis* cannot effectively colonize the roots of *Arabidopsis* plants deficient in soluble sucrose. Sucrose in *Arabidopsis* root exudates triggers a signaling cascade that activates the solid surface motility (SSM) of *Bacillus subtilis*. This activation occurs by inducing bacterial extracellular production of polymeric levan, which in turn facilitates strong synthesis of surfactin and promotes hyper-flagellation of bacteria. Ultimately, this process improves colonization by *Bacillus* [87]. Bacterial surfactin is not only an antibiotic compound but also important for *Bacillus* motility [106]. Surfactin synthesis is promoted by root exudates, thereby enhancing early root colonization by *Bacillus* sp. [107]. Certain plant polysaccharides, the primary components of the plant cell wall, have been shown to enhance the biofilm formation of *B. subtilis* by acting as signals that control the phosphorylation of the master regulator Spo0A and as a carbon resource for producing the matrix exopolysaccharide [88]. Notably, plant polysaccharides also induce bacterial surfactin synthesis, which contributes to the enhancement of root colonization [108]. In addition, glucose, which acts as a carbon resource and a signal for root colonization, is a strong chemoattractant for many fungal strains [91]. Furthermore, ptsG is the major glucose transporter in the beneficial strain *B. cereus* C1L and participates in bacterial colonization in the maize rhizosphere [89].

### 4.4. Sugar Alcohols

Sugar alcohols are polyols imported by secondary active proteins in plants with broad substrate specificity [26]. Inositol, a sugar alcohol, is known as an important eukaryotic structural and signaling molecule [109]. The most abundant inositol isomer present in root exudates is myo-inositol (referred to as inositol in this review) [15]. Recent studies have revealed that inositol can act as a carbon source to promote bacterial growth and as a signaling molecule to enhance various processes, including chemotaxis, biofilm formation, siderophore production, and colonization by beneficial plant-associated bacteria [90,91,92]. In *Arabidopsis*, inositol transporters, such as INT1 and PMT5, are necessary for the secretion of inositol by roots, which mediates the degree of microbial root colonization [92]. DNA methylation is an epigenetic marker in plants that occurs in response to various environmental stimuli and is important for various fundamental biological processes [110]. In addition to inositol transporters in *Arabidopsis*, the root secretion of inositol is regulated by active DNA demethylation, which antagonizes RNA-directed DNA methylation, controls inositol homeostasis gene expression, and subsequently determines efficient colonization by *B. megaterium* YC4 [90]. The enhanced chemotaxis and biofilm formation of YC4 in response to inositol were believed to be due to the inositol-mediated transcriptional regulation of the genes involved in flagellar development, chemotaxis, and biofilm formation [90]. Bacterial inositol catabolism (iol locus) enhances bacterial competence in natural soil and is associated with greater root colonization. For example, recent studies have shown that inositol can enhance swimming motility and growth and alter the colony morphology of *Pseudomonas* and *Pantoea* strains via inositol catabolism [91,92]. In *Pseudomonas* strains, the inositol-induced repression of DksA, a transcriptional regulator involved in inhibiting swimming motility, has been reported as a potential mechanism for inositol-triggered swimming motility [92].

### 4.5. Flavonoids

Flavonoids are particularly important for plant interactions with nitrogen-fixing bacteria and are structurally diverse and tailored to different bacteria [28]. Flavonoids, including the flavanone naringenin and the flavonols quercetin and kaempferol, modulate the diversity of the *Arabidopsis* root microbiota, particularly favoring the recruitment of the family *Aeromonadaceae*. A representative strain, *Aeromonas* sp. H1, which belongs to the *Aeromonadaceae* family, has been shown to increase plant resilience against dehydration. Flavonoids attract the H1 strain by upregulating the transcription of the genes related to flagellum biogenesis and downregulating the fumarate reduction-related genes to promote smooth swimming, and they increase biofilm production [93]. The apigenin and other flavones in rice root exudates enhance biofilm formation by the nitrogen-fixing bacterium *Gluconacetobacter diazotrophicus*. Increased biofilm production further attracts diazotrophic bacteria to the rhizosphere, rhizoplane, and endorhizosphere, thus promoting biological nitrogen fixation [12]. However, maize root-derived flavones, including apigenin and luteolin, predominantly increase the presence of *Oxalobacteraceae* taxa in the soil rhizosphere, which in turn stimulates the growth of lateral roots through the LRT1 signaling pathway and improves maize nitrogen acquisition [94]. Despite the variance in bacterial taxa drawn to flavonoids across different plant rhizospheres, the majority of these attracted bacteria are involved in nitrogen fixation.

### 4.6. Phenolics

Phenolic compounds released by plants can also mediate defense mechanisms against pathogens and attract some microbes by serving as signaling molecules and carbon sources [111]. For example, the increase in the exudation of phenolic compounds, such as vanillin, syringic acid, vanillic acid, and ferulic acid, by *Avena fatua* into the rhizosphere was believed to be a potential mechanism for allelopathy in roots [95]. In addition to improving iron nutrition in plants, various kinds of coumarins, phenolic secondary metabolites of plants, have been shown to have antimicrobial effects [10,41]. For instance, the abundance of several microbes, such as *Pseudomonas* sp. Root329, in the *Arabidopsis* rhizosphere, is inhibited by the coumarins sideretin and fraxetin via redox-mediated antimicrobial mechanisms, which can both mobilize ferric iron and generate ROS [10]. The coumarins fraxetin and scopoletin have selective antimicrobial effects on *Burkholderiaceae* strains and the soil-borne fungal pathogens *Fusarium oxysporum* and *Verticillium dahlia*, respectively [40,41]. However, the growth of the plant-beneficial strains *P. simiae* WCS417 and *P. capeferrum* WCS358, which are highly coumarin-tolerant strains, is not repressed by coumarins. The coumarins in root exudates induced by these plant-associated beneficial bacteria promote their occupation of the rhizosphere niche and subsequently enhance plant growth under iron starvation [10,40,96]. Plant-associated bacteria that can metabolize root-secreted antimicrobial substances exhibit greater rhizosphere competence and successful plant colonization (Figure 1).

### 4.7. Root Volatiles

Root volatiles, including volatile organic compounds (VOCs) and inorganic molecules, play critical roles in long-distance interactions in the soil rhizosphere and serve as energy sources or signaling molecules that regulate bacterial growth, chemotaxis, and competition [112,113]. The sphere of influence of some VOCs attracts bacteria from distances extending beyond the millimeter scale and up to 12 cm away from the roots [59]. Due to their physicochemical properties, root volatiles can easily diffuse throughout the soil, affecting a broad area and impacting microbial communities. The diffusion abilities of root volatiles vary, and the composition and concentration of root volatiles strongly depend on the presence of different microbes. For instance, some VOCs, such as terpenes and terpenoids produced by plants, increase upon pathogen infection, and these induced defensive VOCs inhibit pathogen growth through antimicrobial effects and attract and promote the growth of specific beneficial bacteria through their roles as signals and carbon sources [28,59]. In addition to biotic stress, under nutrient limitation, VOCs, such as terpenes and ketones, can function as infochemicals, providing information about nearby nutrient-rich environments and thereby attracting potentially beneficial bacteria such as *Paenibacillus* [59,114]. VOCs, including terpenes, can reportedly interact with distant soil bacteria and affect their colonization by altering bacterial motility [115]. In addition, some plant VOCs, such as terpenes, can even also act as nutrient sources [59].

Furthermore, not only organic but also inorganic volatiles play a role in shaping microbial communities. Researchers have highlighted the diverse responses of bacteria and fungi to specific inorganic volatiles. For instance, hydrogen (H_2_) was shown to increase the relative abundance of several taxa, such as *Nitrososphaera* and *Gaiella*, in soil bacterial and archaeal communities but had little impact on the fungal community [97]. In contrast to H_2_, in the case of CO_2_, the fungal community in soil has been found to be more susceptible than the bacterial community [116]. However, the mechanisms underlying the diverse responses of different groups of microorganisms to certain root volatiles have not been elucidated.

### 4.8. Other Secondary Metabolites

Secondary metabolites are prevalent in root exudates and serve as signals that promote colonization by certain bacteria. Camalexin and benzoxazinoids are indole-derived specialized secondary metabolites that are important for interactions between rhizobacteria and plants and are involved in plant defenses against pathogens [5]. Additionally, the exudation of camalexin from *Arabidopsis* roots is elicited by pathogens and flagellin, and the accumulation of camalexin can improve the growth-promoting effects of some beneficial strains, such as *Pseudomonas* sp. [98]. Benzoxazinoids are important antimicrobial compounds in many grasses and can be produced in response to pathogen infection or insect damage [99]. Notably, in addition to beneficial bacteria, the exudation of benzoxazinoids also enriches some potential plant pathogenic fungi, but overall, these compounds increase plant defenses and establish a more health-promoting root microbiota [100]. Root exudates, like benzoxazinoids, not only contribute to direct effects from plants on rhizobacteria but also play important roles in indirect impacts by regulating the interactions among rhizosphere signaling molecules. It has been reported that benzoxazinoids can influence the soil rhizobiome through a global regulatory function on plant-derived secondary metabolites in the rhizosphere, especially flavonoids [117]. The interactions among rhizosphere signaling molecules are also important for occupying the rhizosphere niche. Due to the complex interactions among different signals, decoding the clear role of rhizosphere signals in the rhizosphere is challenging. In addition, distinct secondary metabolites attract specific strains that are equipped with corresponding signaling receptors or genes. For instance, the ACC released in root exudates acts as a potent chemoattractant for bacteria harboring the acdS gene, which is integral to the response [14].

## 5. Summary and Future Perspectives

The importance of beneficial rhizobacteria and the process of their root colonization have been well established. Root exudates play a crucial role in shaping the root microbiome. However, the dynamic interaction between root exudates and beneficial rhizobacteria, as well as the specific role of certain root exudates in root colonization by beneficial rhizobacteria, have not been comprehensively concluded. Root exudates are key factors in the selective colonization of the soil rhizosphere by beneficial rhizobacteria. In this review, we summarized the factors influencing root exudate–rhizobacteria interactions, the specific metabolic mechanism through which rhizobacteria affect colonization, and the roles of root exudates in the occupation of rhizosphere niches by beneficial rhizobacteria.

The dynamic nature of root exudation and the soil rhizosphere microbiome is determined by plant genotype, plant development, and environmental abiotic and biotic factors. Root exudates serve multiple functions by acting as nutrients, signals, and even antimicrobial substances that contribute to bacterial colonization. We summarized the roles of several typical and important root exudates in the root colonization of beneficial soil bacteria in recent studies. The multiple functions of root exudation in root colonization by beneficial rhizobacteria are specific and determined by their specific metabolic mechanisms, receptors, and signals. Overall, the interaction between root exudates and beneficial soil bacteria in the rhizosphere is a dynamic process that strongly influences plant growth, nutrient acquisition, and resistance to pathogens (Figure 2). Research advances in the chemistry of root exudates, the mechanisms of microbial recruitment and colonization, and their effects on plant health enhance our ability to harness these interactions for agricultural benefit.

However, many issues are still unresolved. Due to the complex metabolic reactions of soil microorganisms and the chemical complexity of soil, root exudation is traditionally analyzed in hydroponic systems. It is challenging to analyze root exudation in natural environments. Additionally, there are many root exudates that regulate bacterial rhizosphere colonization, but most of the substances in these root exudates are still unknown. Interactions involving plant-associated bacteria and hosts are regulated by appropriate chemical signals, which can occur in a specific or universal manner. However, the specific molecular mechanisms underlying colonization by bacteria in the rhizosphere by diverse root exudates, including the binding mechanisms between substances and bacteria-related receptors and the signaling cascade reactions after bacteria receive substances, are unclear. There are still many questions about the complex network of different signals in root exudates, multiple functions of the same substance in bacterial colonization, and the genetic basis of root–microbe interactions. In addition, the final signaling states of many substances regulating bacterial colonization are still unclear. Do signal substances function in rhizosphere colonization as molecular structures in root exudates or as downstream products of bacteria-specific catabolism systems?

Manipulating root exudation patterns through breeding or biotechnological interventions offers a promising avenue for naturally enhancing beneficial microbial associations. Understanding the mechanisms and factors that govern this interaction can pave the way for developing sustainable and eco-friendly strategies in agriculture and forestry.

## Figures and Tables

**Table 1:** Information of several compounds in root exudates involved in the colonization of beneficial soil rhizobacteria.

Class	Compounds	Host Plants	Strains	Function	Reference
Organic acids	Fumaric acid, tartaric acid, and L-malic acid	Maize	*Hansschlegelia zhihuaiae* S113	Regulate chemotactic response, motility, and biofilm formation	[82]
	Oxalate	Lupin	Plant-associated *Burkholderia* species	Carbon source	[83]
	Citric acid	Rice	*B. altitudinis* LZP02	Chemotaxis, biofilm formation, and the growth-promoting ability	[84]
	Citric, malic, and oxalic acid	Peanut	*Burkholderia pyrrocinia* strain P10	Biofilm formation	[13]
Amino acids	Tryptophan	Cucumber	*B. velezensis* SQR9	Promote colonization and production of indole-3-acetic acid (IAA)	[78]
	Histidine, arginine, and aspartate	Sesbania rostrata	*Azorhizobium caulinodans* ORS571	Chemotaxis	[85]
	Alanine, asparagine, lysine, methionine, and serine	Tomato	*P. fluorescens* Pf0-1	Chemotaxis	[86]
Sugars	Sucrose	*Arabidopsis*	*B. subtilis*	Regulate solid surface motility and colonization	[87]
	Polysaccharides	*Arabidopsis*	*B. subtilis*	Carbon source and biofilm formation signals	[88]
	Glucose	Maize	*B. cereus* C1L	Carbon resource and a signal for root colonization	[89]
Sugar alcohols	Inositol	*Arabidopsis*	*B. megaterium* YC4	Regulate flagellar development, chemotaxis, and biofilm formation	[90]
	Inositol	*Arabidopsis*	*Pseudomonas* and *Pantoea* strains	Enhance swimming motility and growth and alter the colony morphology	[91,92]
Flavonoids	Flavanone naringenin, flavonols quercetin, and kaempferol	*Arabidopsis*	*Aeromonas* sp. H1	Trigger chemotaxis through transcriptionally regulating fumarate reductase and flagellum proteins, and increase biofilm production	[93]
	Apigenin and other flavones	Rice	*Gluconacetobacter diazotrophicus*	Biofilm formation	[12]
	Apigenin and luteolin	Maize	*Oxalobacteraceae* taxa	Enhance bacterial abundance in the rhizosphere	[94]
Phenolics	Syringic acid, vanillin, 4-hydroxybenzoic acid, syringaldehyde, ferulic acid, p-cumaric acid, and vanillic acid	Oat	Rhizosphere microorganisms	Mediate allelopathy	[95]
	Coumarins fraxetin and scopoletin	*Arabidopsis*	*P. simiae* WCS417 and *P. capeferrum* WCS358	Promote their abundance in the rhizosphere but show antimicrobial effects on some other strains	[10,40,96]
Volatiles	Terpenes and ketones	*Carex arenaria*	Specific bacteria with antifungal properties (such as *Paenibacillus sp.*)	Act as nutrients and infochemicals for long-distance interaction, motility, and chemotaxis	[59]
	Hydrogen (H_2_)	Legume	*Nitrososphaera* and *Gaiella*	Enhance bacterial abundance in the rhizosphere	[97]
Other secondary metabolites	ACC	Wheat	Bacteria with ACC deaminase	Nitrogen source, chemoattractant	[14]
	Camalexin	*Arabidopsis*	*Pseudomonas* sp.	Improve the growth-promoting effects of some beneficial strains and show antimicrobial effect on pathogens	[4,98]
	Benzoxazinoids	Maize	Rhizobiome	Increase plant defenses and establish a more health-promoting root microbiota	[99,100]
